# Do invasive plant species profit from pollution with synthetic organic chemicals?

**DOI:** 10.1111/nph.20155

**Published:** 2024-09-27

**Authors:** Yudi M. Lozano, Matthias C. Rillig

**Affiliations:** ^1^ Freie Universität Berlin, Institute of Biology, Plant Ecology D‐14195 Berlin Germany; ^2^ Berlin‐Brandenburg Institute of Advanced Biodiversity Research (BBIB) D‐14195 Berlin Germany

**Keywords:** contamination, environmental tolerance, global change factors, native species, noninvasive species, phenotypic plasticity, plant–soil systems, plant traits

Synthetic organic chemicals are a hallmark of the Anthropocene (Waters *et al*., 2016). The diversity and quantity of synthetic chemicals have surged dramatically, with over 350 000 chemicals and chemical mixtures registered for commercial application world‐wide, the vast majority organic chemicals (Wang *et al*., 2020). Given this, it has been argued that synthetic chemicals are agents of global change (Bernhardt *et al*., 2017) with different potential effects on terrestrial systems. Specifically, synthetic organic chemicals are increasingly recognized as an important threat to biodiversity and ecosystems (Sigmund *et al*., 2023). Fertilizers, pesticides, surfactants, persistent organic pollutants, pharmaceutical and personal care products, per‐ and polyfluoroalkyl substances (PFAS), micro/nano plastics, organic solvents, antibiotics, endocrine‐disrupting compounds, engineered nanoparticles, food and feed additives, among others, are widely employed in manufacturing, medicine and agriculture. These substances pollute terrestrial ecosystems via industrial discharges, spills, leaks, agricultural runoff, household products, and improper waste disposal (Wang *et al*., 2024).

Synthetic organic chemicals exert negative effects on soil biota and plant performance. For instance, pesticides are produced to prevent the growth of unwanted organisms such as weeds or pathogens. However, they also kill or affect the reproductive potential of nontarget organisms like beneficial nematodes or earthworms with negative consequences on plant–soil systems (Ankit *et al*., 2020). However, could some plants profit from this widespread pollution with synthetic organic chemicals? While various plant species might take advantage of this new situation, invasive species, with their set of characteristics, seem particularly well‐suited. We consider that the diversity of these pollutants, their varying concentration, and their world‐wide distribution create numerous novel environments from which invasive species could potentially benefit.

What would be the different mechanisms that may enable plant invasiveness in chemically polluted soils? (Fig. 1). Different competitive characteristics can enable certain alien or native plants to become invasive, and these traits may also contribute to a plant's ability to tolerate polluted environments. For example, traits related to growth rate, physiology, size, and fitness – such as rapid growth – are commonly observed in invasive species and could similarly enhance a plant's capacity to survive in polluted soils. However, we acknowledge that this relationship is not universally applicable to all invasive plants (van Kleunen *et al*., 2010b). While there may be some overlap, the presence of these traits does not necessarily imply pollution tolerance across all species.

**Fig. 1:**
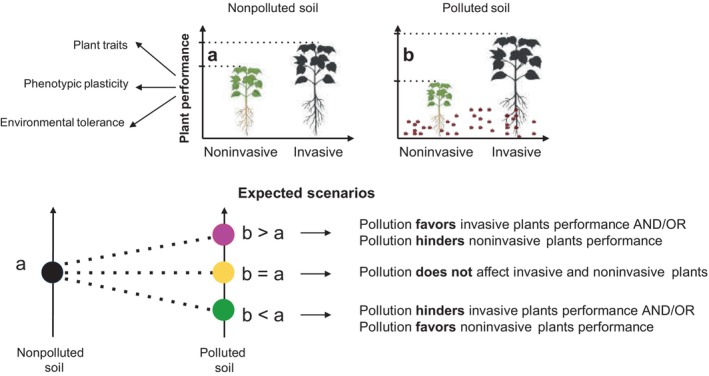
Possible mechanisms by which soil pollution may promote invasive plant species. Pollution with synthetic organic chemicals could promote the relative performance of invasive species over noninvasive ones, since the former may possess traits, phenotypic plasticity and environmental tolerance that allow them to thrive in polluted environments. This advantage can lead to their dominance within the plant community. Depending on the performance difference between noninvasive and invasive plants, there are three scenarios. Here, we propose that invasive plants are favored by pollution (b > a).

Plant invasion is often driven by specific traits, leading to high dominance within the community. However, the mechanisms underlying plant invasion in the context of soil chemical pollution remain largely unexplored. Invasive species can possess advantageous traits that might enable them to avoid or better tolerate chemical pollution, potentially making them less susceptible to pollutants than their noninvasive counterparts. Plants can invade vegetation in various ways, likely relying on different traits to do so (Lai *et al*., 2015). For instance, invasive species might have favorable root traits making them less susceptible to chemical pollution. Roots are often the first tissue that comes in contact with soil pollutants, and therefore absorption via roots is the most common method of uptake. Invasive species often exhibit faster and greater biomass allocation to roots compared to native species (van Kleunen *et al*., 2010; Keser *et al*., 2014). Indeed, invasive species have been observed to develop deep roots in polluted roadside environments (Brisson *et al*., 2010), which potentially allows them to avoid stress from contamination by accessing deeper soil layers that are less affected by pollutants or by extending their roots laterally. Likewise, invasive species can produce thicker roots that are often less absorptive, which appears to contribute to their competitive advantage over noninvasives in polluted soils (Fu *et al*., 2022). This would help them reduce the sorption of soil pollutants, thus reducing their exposure to pollutants in soil. Likewise, invasive species could also be able to escape from chemical pollution due to phytovolatilization. That is, invasive species can uptake contaminants from the soil and subsequently convert and release them as less toxic vapors into the atmosphere through transpiration (Prabakaran *et al*., 2019). Poplar trees, invasive plants in South Africa (Robinson *et al*., 2017), were capable of taking up trichloroethylene, an industrial halocarbon, and phytovolatilizing it in measurable amounts in its introduced range (Newman *et al*., 1997). By doing so, invasive species can reduce their own exposure to toxic substances, partially escaping from chemical pollution. Such a phenomenon is also plausible in its native range (Strycharz & Newman, 2009), and it is thus an example of a plant trait that may provide additional benefit in polluted areas outside of the native range of the plant. While this specific trait may not be common in many invasive species, the presence of such plants could increase local richness, increasing the likelihood of finding unique traits. Additionally, due to the relatively low‐host specificity of arbuscular mycorrhizal fungi (AMF), AM symbioses could support the success of invasives such as the shrub *Corchotus capsulari*; besides improving plant nutrition, AMF may lower soil anthracene concentrations by boosting root oxidoreductase activity (Cheung *et al*., 2008).

Other traits such as height, specific leaf area, seed mass, and reproductive type have a complex relation with plant invasion as they can show positive, negative, or no relationship across different invasion dimensions such as spread rate, local abundance, or geographic and environmental range sizes (Palma *et al*., 2021). However, the specific response of these traits in chemically polluted soils needs to be evaluated. For instance, invasive plants with large seeds (Daws *et al*., 2007), may escape from pollutants and maintain better germination rates than noninvasive species with typically smaller seeds. This is because larger seeds normally have larger endosperms, which serve as energy reserves for germination (Valencia‐Díaz *et al*., 2015), allowing these plants to initially depend less on external resources. Additionally, the thicker seed coat helps reduce water permeability (Souza & Fagundes, 2014), which together, may help seeds to avoid pollutants. By contrast, invasive species with smaller seeds can increase their chances of germination, potentially even in polluted soils (Lake & Leishman, 2004), or avoid these pollutants by dispersing over large distances.

Invasive species can possess antioxidant systems that potentially could mitigate the oxidative stress induced by chemical pollution, as these antioxidants offer superior protection against stress (Pintó‐Marijuan & Munné‐Bosch, 2013). For instance, the invasive forb *Wedelia trilobata* has greater tolerance to the antibiotic Levofloxacin hydrochloride compared to its native noninvasive counterpart *Wedelia chinensis*, largely due to its robust antioxidant system (Huang *et al*., 2023). Indeed, invasive plants often exhibit broad environmental tolerance (Moura *et al*., 2021), which may allow them to thrive in polluted soils. For instance, invasive aquatic plants have shown greater tolerance to the antibiotic ciprofloxacin compared to noninvasive plants (Fan *et al*., 2024). Additionally, populations of invasive plants evolve greater tolerance to biotic and abiotic stressors (Gong *et al*., 2022). This may suggest evolving a greater tolerance to synthetic pollutants, which in the end helps to promote plant invasiveness. Importantly, the mechanisms behind this process for such specific stressors are still unknown.

Phenotypic plasticity, the ability of a plant to manifest varying phenotypes in response to different environmental conditions (Bradshaw, 1965), enhances the invasiveness of plants, aiding their spread and establishment in new environments (Zenni *et al*., 2014). Invasive species exhibit higher phenotypic plasticity compared to noninvasive species (Davidson *et al*., 2011). However, such a response depends on the environmental stressor and the phenotypic trait (Zhang *et al*., 2023). Thus, whether invasive plants exhibit higher phenotypic plasticity that noninvasives to chemically polluted soils may be evaluated based on the trait and the specific pollutant.

We explored potential traits through which invasive plants might outcompete noninvasive species in soils polluted with synthetic organic chemicals. We highlighted some common traits shared by various invasive plants, but the literature shows that invasive species can have a wide variety of traits, in many cases contrasting. Therefore, clearly more studies are needed to test if such effects hold when a greater variety of cases (in terms of plants, traits, and pollutants) are considered. Species with these traits could positively impact heavily polluted soils such as lead and zinc mining areas or toxic waste dumps, where vegetation is absent and unlikely to flourish. In such environments, species of invasive character may act as early colonizers rather than being viewed as invaders, helping to initiate the recovery of these areas. However, to use them effectively, it is crucial to control their spread beyond these specific soils to avoid them from dominating surrounding plant communities. Therefore, thorough research is essential before considering the use of invasive species for this purpose.

Overall, we foresee the need for more experimental work, for tapping existing databases and for targeted observational studies. A priority should be conducting toxicity assays using individual chemicals, in mixture, and at different concentrations, to assess the potential competitive advantage of invasive species over noninvasive in terms of root development, reproductive capacity, and mycorrhizal associations, among other plant traits. Collating already existing data on invasive plant traits – such as root growth, exudates, seed germination, offspring survival, AMF structures – along with bioaccumulation studies on plant tissues, will help predict invasiveness patterns in polluted soils. Factorial experiments can be performed to assess the potential synergistic or antagonistic effects between synthetic organic chemicals and/or in combination with other global change drivers, such as drought and temperature. Importantly, variables such as high‐nutrient availability or disturbance could covary with high‐pollutant concentrations. Therefore, experiments should be designed to avoid these confounding factors and distinguish them from the direct effects of pollutants as a potential causal effect promoting plant invasiveness. Observational studies can be used to find patterns linking soil pollution with invasive plant success. Many of these studies can be performed in the invasive range of the plants, but experiments should also test whether there is a difference compared to plant performance in their native range, in case enhanced performance under pollution has evolved in the invaded range. Additionally, identifying whether a species is invasive or noninvasive can be complex. Thus, in agreement with Palma *et al*. (2021), we consider it to be more informative to study the dimensions of invasiveness, such as the rate of spread, environmental and geographic range sizes, and relative local abundance (i.e. dominance) of the plants, rather than relying on binary classifications.

Plant invasion and soil pollution by synthetic organic chemicals are currently mostly tackled separately by different scientific groups. In agreement with Sigmund *et al*. (2023), we suggest that success in addressing these issues requires collaboration across disciplines, including ecology, ecotoxicology, and environmental chemistry. While we need a more comprehensive analysis of the effects of synthetic organic pollutants on the full range of plant traits and life history characteristics of our plant communities, focusing initially on invasive plants is crucial. If increasing soil pollution with synthetic organic chemicals indeed broadly favors invasive species, then we have been underestimating the true effects of such substances on the environment.

## Competing interests

None declared.

## Author contributions

YML and MCR contributed equally to this work.

## References

[nph20155-bib-0001] Ankit SL , Kishor V , Bauddh K . 2020. Impacts of synthetic pesticides on soil health and non‐targeted Flora and Fauna. In: Bauddh K , Kumar S , Singh RP , Korstad J , eds. Ecological and practical applications for sustainable agriculture. Springer: Singapore, Singapore, 65–88.

[nph20155-bib-0002] Bernhardt ES , Rosi EJ , Gessner MO . 2017. Synthetic chemicals as agents of global change. Frontiers in Ecology and the Environment 15: 84–90.

[nph20155-bib-0003] Bradshaw AD . 1965. Evolutionary significance of phenotypic plasticity in plants. In: Caspari EW , Thoday JM , eds. Advances in genetics. New York, NY, USA and London, UK: Academic Press, 115–155.

[nph20155-bib-0004] Brisson J , De Blois S , Lavoie C . 2010. Roadside as invasion pathway for common reed (*Phragmites australis*). Invasive Plant Science and Management 3: 506–514.

[nph20155-bib-0005] Cheung KC , Zhang JY , Deng HH , Ou YK , Leung HM , Wu SC , Wong MH . 2008. Interaction of higher plant (jute), electrofused bacteria and mycorrhiza on anthracene biodegradation. Bioresource Technology 99: 2148–2155. 17662599 10.1016/j.biortech.2007.05.037

[nph20155-bib-0006] Davidson AM , Jennions M , Nicotra AB . 2011. Do invasive species show higher phenotypic plasticity than native species and, if so, is it adaptive? A meta‐analysis: Invasive species have higher phenotypic plasticity. Ecology Letters 14: 419–431. 21314880 10.1111/j.1461-0248.2011.01596.x

[nph20155-bib-0007] Daws MI , Hall J , Flynn S , Pritchard HW . 2007. Do invasive species have bigger seeds? Evidence from intra‐ and inter‐specific comparisons. South African Journal of Botany 73: 138–143.

[nph20155-bib-0008] Fan P , Yu H , Lv T , Wang H , Li D , Tong C , Wu Z , Yu D , Liu C . 2024. Alien emergent aquatic plants develop better ciprofloxacin tolerance and metabolic capacity than one native submerged species. Science of the Total Environment 932: 173030. 38719043 10.1016/j.scitotenv.2024.173030

[nph20155-bib-0009] Fu Y , van Kleunen M , Ma K , Liu Y . 2022. The more synthetic polymer types pollute the soil, the stronger the growth suppression of invasive alien and native plants. *bioRxiv* . doi: 10.1101/2022.12.08.519663.

[nph20155-bib-0010] Gong W , Wang Y , Chen C , Xiong Y , Zhou Y , Xiao F , Li B , Wang Y . 2022. The rapid evolution of an invasive plant due to increased selection pressures throughout its invasive history. Ecotoxicology and Environmental Safety 233: 113322. 35182800 10.1016/j.ecoenv.2022.113322

[nph20155-bib-0011] Huang P , Wang H , Xu Z , Yang H , Du Y , Chen S , Abbas A , Yin H , Sun P , Du D . 2023. The responses of invasive *Wedelia trilobata* and native *Wedelia chinensis* to levofloxacinhydrochloride: implication for biological invasion. Polish Journal of Environmental Studies 32: 3605–3615.

[nph20155-bib-0012] Keser LH , Dawson W , Song Y‐B , Yu F‐H , Fischer M , Dong M , van Kleunen M . 2014. Invasive clonal plant species have a greater root‐foraging plasticity than non‐invasive ones. Oecologia 174: 1055–1064. 24352844 10.1007/s00442-013-2829-y

[nph20155-bib-0014] van Kleunen M , Weber E , Fischer M . 2010. A meta‐analysis of trait differences between invasive and non‐invasive plant species. Ecology Letters 13: 235–245. 20002494 10.1111/j.1461-0248.2009.01418.x

[nph20155-bib-0015] Lai HR , Mayfield MM , Gay‐des‐combes JM , Spiegelberger T , Dwyer JM . 2015. Distinct invasion strategies operating within a natural annual plant system. Ecology Letters 18: 336–346. 25728390 10.1111/ele.12414

[nph20155-bib-0016] Lake JC , Leishman MR . 2004. Invasion success of exotic plants in natural ecosystems: the role of disturbance, plant attributes and freedom from herbivores. Biological Conservation 117: 215–226.

[nph20155-bib-0017] Moura RF , Queiroga D , Vilela E , Moraes AP . 2021. Polyploidy and high environmental tolerance increase the invasive success of plants. Journal of Plant Research 134: 105–114. 33155178 10.1007/s10265-020-01236-6

[nph20155-bib-0018] Newman LA , Strand SE , Choe N , Duffy J , Ekuan G , Ruszaj M , Shurtleff BB , Wilmoth J , Heilman P , Gordon MP . 1997. Uptake and biotransformation of trichloroethylene by hybrid poplars. Environmental Science & Technology 31: 1062–1067. 10.1289/ehp.98106s41001 PMC1533336 9703485

[nph20155-bib-0019] Palma E , Vesk PA , White M , Baumgartner JB , Catford JA . 2021. Plant functional traits reflect different dimensions of species invasiveness. Ecology 102: e03317. 33638164 10.1002/ecy.3317

[nph20155-bib-0020] Pintó‐Marijuan M , Munné‐Bosch S . 2013. Ecophysiology of invasive plants: osmotic adjustment and antioxidants. Trends in Plant Science 18: 660–666. 24001766 10.1016/j.tplants.2013.08.006

[nph20155-bib-0021] Prabakaran K , Li J , Anandkumar A , Leng Z , Zou CB , Du D . 2019. Managing environmental contamination through phytoremediation by invasive plants: a review. Ecological Engineering 138: 28–37.

[nph20155-bib-0023] Robinson T , Ivey P , Powrie L , Winter P , Wong LJ , Shyama Pagad . 2017. Global Register of Introduced and Invasive Species – South Africa: 2107 records. Invasive Species Specialist Group ISSG

[nph20155-bib-0024] Sigmund G , Ågerstrand M , Antonelli A , Backhaus T , Brodin T , Diamond ML , Erdelen WR , Evers DC , Hofmann T , Hueffer T *et al*. 2023. Addressing chemical pollution in biodiversity research. Global Change Biology 29: 3240–3255. 36943240 10.1111/gcb.16689

[nph20155-bib-0025] Souza ML , Fagundes M . 2014. Seed size as key factor in germination and seedling development of *Copaifera langsdorffii* (Fabaceae). American Journal of Plant Sciences 5: 2566–2573.

[nph20155-bib-0026] Strycharz S , Newman L . 2009. Use of native plants for remediation of trichloroethylene: II. Coniferous trees. International Journal of Phytoremediation 11: 171–186. 28133996 10.1080/15226510802378459

[nph20155-bib-0027] Valencia‐Díaz S , Flores‐Morales A , Flores‐Palacios A , Perea‐Arango I . 2015. How does the presence of endosperm affect seed size and germination? Botanical Sciences 93: 783–789.

[nph20155-bib-0028] Wang F , Xiang L , Sze‐Yin Leung K , Elsner M , Zhang Y , Guo Y , Pan B , Sun H , An T , Ying G *et al*. 2024. Emerging contaminants: a one health perspective. The Innovations 100612: 100612. 10.1016/j.xinn.2024.100612 PMC11096751 38756954

[nph20155-bib-0029] Wang WGW , Muir DCG , Nagatani‐Yoshida K . 2020. Toward a global understanding of chemical pollution: a first comprehensive analysis of national and regional chemical inventories. Environmental Science & Technology 54: 2575–2584. 31968937 10.1021/acs.est.9b06379

[nph20155-bib-0030] Waters CN , Zalasiewicz J , Summerhayes C , Barnosky AD , Poirier C , Gałuszka A , Cearreta A , Edgeworth M , Ellis EC , Ellis M *et al*. 2016. The Anthropocene is functionally and stratigraphically distinct from the Holocene. Science 351: aad2622. 26744408 10.1126/science.aad2622

[nph20155-bib-0031] Zenni RD , Lamy J‐B , Lamarque LJ , Porté AJ . 2014. Adaptive evolution and phenotypic plasticity during naturalization and spread of invasive species: implications for tree invasion biology. Biological Invasions 16: 635–644.

[nph20155-bib-0032] Zhang X , Oduor AMO , Liu Y . 2023. Invasive plants have greater growth than co‐occurring natives in live soil subjected to a drought‐rewetting treatment. Functional Ecology 37: 513–522.

